# The adverse effects of non-invasive high-frequency oscillatory ventilation on the cerebral hemodynamics of premature infants: point-of-care brain ultrasound findings from a case-series study

**DOI:** 10.3389/fped.2026.1830897

**Published:** 2026-05-20

**Authors:** Jing Liu, Wei Fu, Ying Liu

**Affiliations:** 1 Department of Neonatology and NICU, Beijing Obstetrics and Gynecology Hospital, Capital Medical University, Beijing Maternal and Child Health Care Hospital, Beijing, China; 2 Department of Neonatology and NICU, Beijing Chaoyang District Maternal and Child Healthcare Hospital, Beijing, China

**Keywords:** cerebral hemodynamics, conventional invasive mechanical ventilation, invasive high-frequency oscillatory ventilation, non-invasive high-frequency oscillatory ventilation, noninvasive ventilation, premature infant, respiratory distress

## Abstract

**Objective:**

In recent years, non-invasive high-frequency oscillatory ventilation (NHFOV) has been widely used in clinical practice for premature infants with respiratory distress. However, until now, the focus of all published studies has been on the effect of NHFOV on patient's respiratory system, and its impact on the neonatal cerebral hemodynamics has not been considered.

**Method:**

We monitored the hemodynamics of the anterior cerebral arteries of 10 premature infants who received NHFOV treatment because of respiratory distress and compared them with the cerebral hemodynamics of patients who received invasive high-frequency oscillatory ventilation (IHFOV), invasive conventional mechanical ventilation (IMV), nasal continuous positive airway pressure (NCPAP), and those who did not receive ventilator treatment at the same time.

**Results:**

The results of this study revealed that NHFOV can have serious adverse effects on the cerebral hemodynamics of infants and subsequently lead to brain damage. Most importantly, this adverse effect of NHFOV is even more severe than that caused by IHFOV and IMV.

**Conclusion:**

Given that NHFOV has a severely adverse effect on the cerebral hemodynamics of patients compared with other non-invasive ventilation modes, it does not have any distinct advantages; therefore, we believe that NHFOV should be used with caution in premature infants, especially those who are extremely premature.

## Introduction

In recent years, non invasive high-frequency oscillatory ventilation (NHFOV) has been widely studied and applied in neonatal clinical practice, especially in the respiratory management of premature infants. It is believed that compared with other noninvasive ventilation modes, NHFOV has the same therapeutic effect, does not increase the risk of complications, and may even be advantageous for reducing the risk of reintubation after extubation in premature infants (1–5). However, these studies focused only on the impact of NHFOV on the respiratory system and overlooked its effects on other organs, particularly its effects on neonatal cerebral hemodynamics. Previous studies have shown that different mechanical ventilation methods, including non- invasive ventilation (such as nasal continuous positive airway pressure (NCPAP)) or invasive conventional (such as synchronized intermittent ventilation (SIMV) and high-frequency ventilation (HFV)) approaches, have a serious adverse effect on the cerebral hemodynamics of newborns and premature infants and are closely related to intracranial hemorrhage and periventricular white matter injury (PWI) in premature infants (6, 7). The specialized critical care ultrasound technique used in our department has been applied to the management of all critically ill patients. While lung ultrasound (LUS) is used to guide the management of lung diseases, bedside critical care ultrasound technology is also employed to guide the management of critically ill children (8, 9). Therefore, in addition to monitoring respiratory system-related clinical manifestations and changes in arterial blood gas measurements in infants during treatment with NHFOV, we also monitored changes of cerebral hemodynamic parameters because we are concerned that NHFOV might also have a severely adverse effect on the cerebral hemodynamics of the patients. This article reports the results of our study on a small sample case series.

## Methods

### Participants

Since the safety of NHFOV for the brain remains unclear, this study selected infants with a gestational age of ≥28 weeks and a birth weight of ≥1,000 g as the research subjects. The control group consisted of children who were hospitalized during the same period and who received conventional invasive mechanical ventilation (IMV), invasive high-frequency oscillatory ventilation (IHFOV) or NCPAP as their treatments. Each ventilation mode involved 10 infants in controls, all of whom had similar gestational ages and birth weight. The written informed consent was obtained from the parents.

### Cerebral hemodynamic monitoring

During the study, all the patients underwent routine cranial ultrasound scans including B-mode and Doppler modalities within 2–4 h after the infants underwent mechanical ventilation treatment. Both the results of routine cranial ultrasound monitoring and the cerebral hemodynamic frequency spectrums of each patient were recorded and preserved. The major results of routine cranial ultrasound examinations included intracranial hemorrhage, PWI, cerebral infection, etc., and both the dynamic and static hemodynamic frequency spectrums were recorded.

## Results

### Demographics and clinical information of the NHFOV patients

Among the 10 premature infants who completed the study, 6 were boys and 4 were girls. The gestational age ranged from 28 to 30 weeks for 5 patients, 31–32 weeks for 3 patients, and 33–35 weeks for 2 patients. The birth weights ranged from 1,000 to 2,200 grams. The time period for cerebral blood flow monitoring ranged from 5 h after birth to 6 days after birth. Based on the clinical manifestations and lung ultrasound findings, 6 patients were diagnosed with RDS, 3 with severe pneumonia (intrauterine infection), and 1 with severe transient tachypnea of the newborn (10–13).

#### Results of cerebral hemodynamic monitoring

All 10 premature infants had severe cerebral hemodynamic disorders, including (1) Abnormal blood flow spectrum morphology was detected: the blood flow spectrum was not smooth, and irregular small wave peaks appeared in both the systolic and diastolic phases. (2) Color Doppler ultrasound revealed cerebral hemodynamic disorders.

#### Results of the brain ultrasound examination

**Figure 1:**
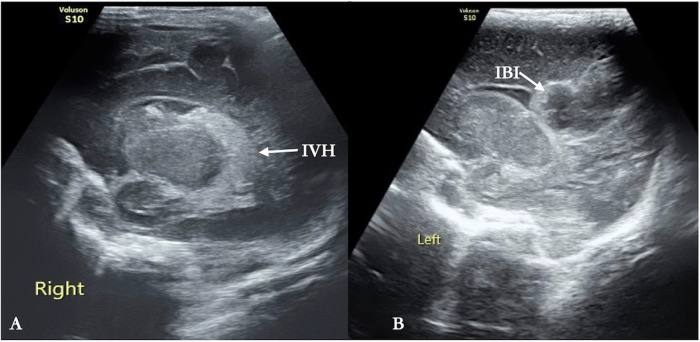
Brain injury. **(A)** The lateral ventricle is filled with echogenic masses, indicating the presence of intraventricular hemorrhage (grade III) on this side of the ventricle (arrows). **(B)** A reflection of an elliptical abnormal echo is clearly demarcated within the cerebral parenchyma of the lateral ventricle body (parietal lobe), suggesting ischemic brain damage in this part of the cerebral parenchyma (arrows).

Among the 10 premature infants, 7 had no obvious abnormalities in the cranial B-mode ultrasound examination, but the remaining 3 had severe abnormalities, including 1 with intraventricular hemorrhage and 1 with an ischemic lesion in the periventricular white matter (Figures 1A,B).

### Typical case representations

#### General information of the patient

A female infant with a gestational age of 29 weeks and a birth weight of 1,450 g was admitted to the hospital because of RDS. After 12 h of IHFOV, LUS revealed that the signs of RDS had disappeared (10, 13). Therefore, the invasive ventilator was removed, and NHFOV-assisted breathing was adopted.

#### Cerebral hemodynamic spectrum of the patient

**Figure 2:**
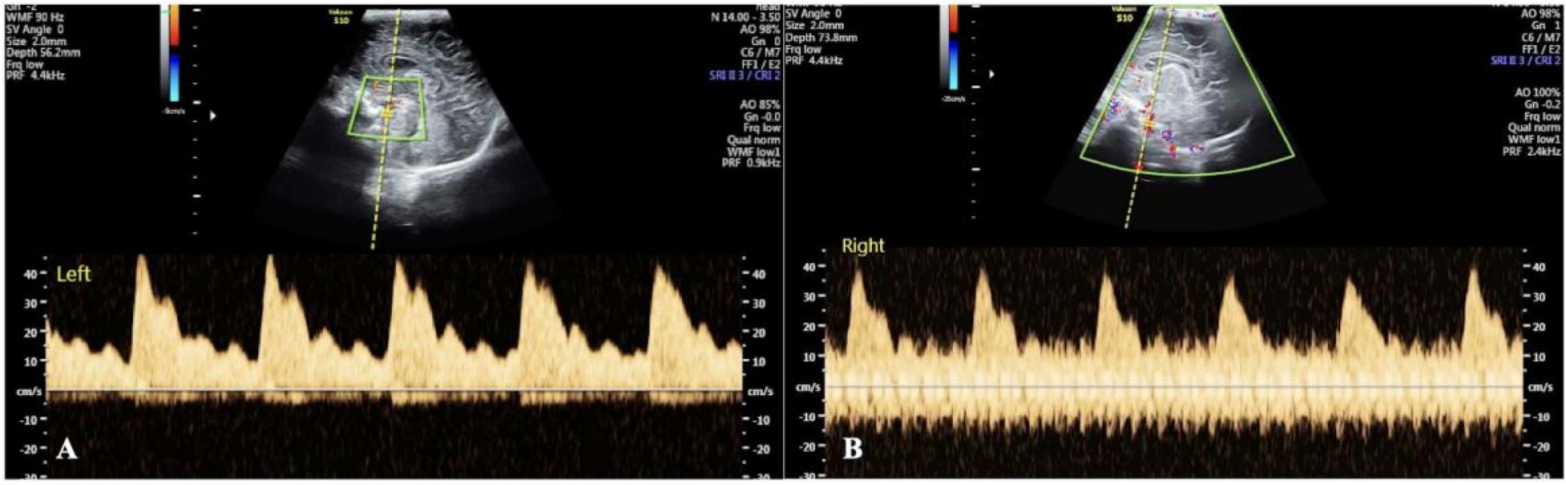
Effect of HOFV on the cerebral hemodynamics of newborns. Both IHFOV **(A)** and NHFOV **(B)** have severe adverse effect on the cerebral hemodynamics of newborns. A serrated small wave appears in both the systolic and diastolic blood flow spectra. However, the impact of NHFOV on cerebral blood flow dynamics is much more significant.

**Figure 3:**
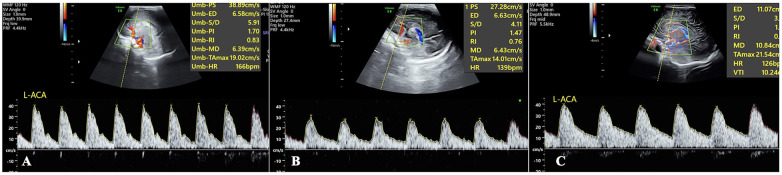
Cerebral blood flow spectrum in patients who received conventional ventilation. The spectra in IMV **(A)** and NCPAP **(B)** patients show a few small, serrated waveforms. However, this abnormality was significantly less severe than that associated with high-frequency ventilation (Figure 2), and its cerebral blood flow spectral pattern was similar to that of patients who did not use a ventilator **(C).**

Both IHFOV and NHFOV caused significant cerebral blood flow disorders, manifested as abnormal spectral patterns. Small sawtooth waveforms can be observed in the blood flow spectra during both systole and diastole, and NHFOV caused more severe abnormalities (Figures 2A,B, Supplementary Videos 1, 2). NCPAP and IMV did not significantly affect cerebral hemodynamic parameters. The cerebral blood flow spectrum presented a regular pattern without jagged waveforms, similar to that of patients who did not receive ventilator treatment (Figures 3A–C). Real-time Doppler ultrasound revealed that the blood flow in the cerebral arteries was essentially normal, and the vascular pathways were clearly visualized, which was similar to the cerebral hemodynamic manifestations of infants who did not receive mechanical ventilation (Supplementary Videos 3–5).

## Discussion

NHFOV has been widely studied and applied in neonatal clinical settings in recent years, and the findings mostly indicate that it has the same effect as other noninvasive modes and does not increase the risk of complications (1–3); some studies have revealed that NHFOV can help reduce the probability of reintubation (4, 5), but more studies have indicated that the effects of the two are comparable (1–3, 14). A recent literature review revealed that compared with NIPPV and NCPAP, NHFOV, as a post-extubation respiratory support method for preterm infants, did not increase the rates of long-term respiratory morbidities or neurodevelopmental impairments (15). However, it has no other significant advantages, and the literature indicates that NHFOV may increase the partial pressure of carbon dioxide in the blood of infants (16). Importantly, to date, no studies on NHFOV have investigated its impact on patients' cerebral hemodynamic parameters.

All types of mechanical ventilation can lead to or increase the incidence of brain injury and cerebral hemodynamic disorders in patients (6, 7). Patients who receive IHFV have a higher incidence of cerebral hemorrhage, which may be related to the abnormal cerebral hemodynamic changes caused by this treatment (17, 18). Conventional cranial ultrasound in this study revealed that among the 10 premature infants, 3 of them had brain injuries (2 of them had intracranial hemorrhage and 1 of them had a cerebral ischemic lesion) (Figure 1), with an incidence rate (30%) much higher than that of brain injuries in patients of the same gestational age (17.8%) during the study period in our NICU. This cannot be ruled out as being related to the cerebral hemodynamic disorders caused by NHFOV.

Notably, owing to the severe disorder and inaccuracy of the spectrum, the accurate measurements of parameters such as the blood flow velocity and resistance index of the spectrum are difficult. Therefore, this study did not measure or statistically analyze the specific cerebral hemodynamic parameters of the infants. Even so, the shape of the spectrum itself is sufficient to confirm the effects of different ventilation modes on patients' cerebral hemodynamics.

Because of our research findings indicate that NHFOV can have severe adverse effects on the cerebral hemodynamics of premature infants. Therefore, after conducting a preliminary study on 10 consecutive infants, we terminated this research and no longer used the NHFOV technique in clinical practice. To draw clinician's attention to this serious issue, this article presents an illustrative case of the adverse effects of NHFOV on the cerebral hemodynamics of patients.

## Conclusion

In conclusion, we believe that compared with traditional noninvasive ventilation, NHFOV does not have any significant advantages and can cause severe cerebral hemodynamic abnormalities and brain damage. Therefore, we believe that NHFOV should be used with caution in premature infants, especially those who are extremely premature.

The main limitation of this study is the small sample size, which is a protective measure to avoid causing further harm to the infants. Furthermore, because high-frequency ventilation causes instability in the cerebral blood flow spectrum, it makes it difficult to measure the specific data of cerebral blood flow dynamic parameters. Although the impact of high-frequency vibration on cerebral blood flow does exist objectively, for these small wave peaks, we need to rule out the possibility of artifacts caused by the high-frequency vibration. Additionally, this article did not focus on the impact of lung diseases on the cerebral hemodynamics in patients. Therefore, in any case, this technique should be used with caution in clinical practice.

## Data Availability

The original contributions presented in the study are included in the article/Supplementary Material, further inquiries can be directed to the corresponding author.

## Supplementary material

Supplementary Videos 1–5 We can see from Videos 1 to 5 that HFOV has a significant influence on cerebral blood flow fluctuations in newborn infants. The harm associated with NHFOV (Video 2) is more significant than that associated with IHFOV (Video 1). NCPAP (Video 4) and IMV (Video 3) have relatively small influences on cerebral blood flow fluctuations, and compared with infants who did not use mechanical ventilation, there was no significant difference in cerebral blood flow (Video 5).
